# Antimicrobial activity of silver free powder coatings based on biocomponents

**DOI:** 10.1038/s41598-025-30166-3

**Published:** 2025-12-02

**Authors:** Katarzyna Krawczyk, Barbara Pilch-Pitera, Michał Kędzierski, Małgorzata Zubielewicz, Ewa Langer, Sebastian Jurczyk, Grażyna Kamińska-Bach, Katarzyna Daszykowska, Leszek Komorowski, Katarzyna Bieniek, Marta Przybysz-Romatowska, Michael Hilt

**Affiliations:** 1 Fraunhofer Institute for Manufacturing Engineering and Automation IPA, Pigments and Coatings Allmandring 37, 70569 Stuttgart, Germany; 2 Faculty of Chemistry, Department of Polymers and Biopolymers, Rzeszow University of Technology, ul. Powstancow Warszawy 6, Rzeszow, 35-029 Poland; 3 Łukasiewicz Research Network – Industrial Chemistry Institute, ul. Ludwika Rydygiera 8, Warsaw, 01-793 Poland; 4 Łukasiewicz Research Network, Institute of Polymer Materials, M. Skłodowskiej- Curie 55, Torun, 87-100 Poland; 5 Road and Bridge Research Institute, ul. Instytutowa 1, Warsaw, 03-302 Poland

**Keywords:** ε-polylysine, Montmorillonite, Antimicrobial activity, Epoxy powder coatings, Bio-based additives, Chemistry, Materials science

## Abstract

In this work, silver-free, low-temperature-curing epoxy powder coatings with antimicrobial functionality were developed. The cationic biopolymer ε-polylysine (PLY) in its protonated form, as well as its intercalation and co-intercalation products with aminododecanoic acid (ADA) in montmorillonite (MMT), were employed as environmentally friendly bioactive additives. The powder coatings were formulated using epoxy resin and a highly reactive phenolic curing agent and subsequently applied onto steel substrates. The resulting materials were comprehensively characterized in terms of surface morphology, gloss, hardness, adhesion, and wettability. The coating showed a water contact angle of approximately 85°and an antibacterial reduction exceeding 99% against *E. coli* and *S. aureus* under laboratory conditions (ISO 22196). Antimicrobial activity is most likely associated with electrostatic interaction between the cationic PLY and bacterial membranes. Overall, this study suggests a promising and sustainable, silver-free strategy for developing antimicrobial powder coatings that may be suitable for hygiene sensitive environments, pending further long-term and safety evaluation.

**Supplementary Information:**

The online version contains supplementary material available at 10.1038/s41598-025-30166-3.

## Introduction

 Polymeric materials with antimicrobial properties are increasingly applied in healthcare, cosmetics, public facilities, and the food industry^1–4^. However, concerns remain regarding the long-term efficacy and environmental safety of biocidal additives. The main challenges include achieving selectivity without affecting non-target organisms, minimizing bioaccumulation, and combating antibiotic-resistant bacteria (“superbugs”)^5^.

Antimicrobial coatings are typically classified as biocide-releasing, contact-active, or anti-adhesive, and combinations of these mechanisms are often employed to enhance overall performance^6,7^. In a comparative study of 23 commercial antimicrobial coatings, Mölling et al.^8^ reported that more than half contained nanosilver and achieved log reductions of approximately six against *E. coli* according to ISO 22,196. Comparable results were obtained for coatings incorporating silver-TiO₂-MMT, zinc-MMT, TiO₂, covalently bound quaternary ammonium compounds (QACs), nanocurcumin/nanoclay systems, triclosan, and zinc pyrithione^9–11^.

The antimicrobial mechanism of silver is primarily attributed to Ag⁺ ions that bind to electron-donating groups in biomolecules such as membrane proteins and enzymes^12^. Despite its strong antimicrobial efficacy, silver exhibits several disadvantages, including high cost, limited availability, potential for bioaccumulation, and the promotion of bacterial resistance^13,14^. In addition, nanosilver particles may induce immunotoxic and inflammatory responses^15,16^. In coatings applications, the use of silver is further restricted by its sensitivity to high curing temperatures and tendency to cause yellowing. To overcome these limitations, binary (Ag⁺/Cu²⁺) and ternary (Ag⁺/Cu²⁺/Zn²⁺) systems have been developed^17,18^, as well as combinations with organic antimicrobial agents such as quaternary ammonium compounds (QACs) and imidazole derivatives^19,20^.

Quaternary ammonium compounds (QACs, R₁R₂R₃R₄N⁺X⁻) exert their antimicrobial effect through a dual mechanism: a hydrophobic alkyl chain that disrupts microbial membranes and a positively charged ammonium group that interacts with negatively charged cells surfaces^21^. These compounds are effective against a broad range of bacteria and enveloped viruses^22,23^. However, microbial resistance to simple and widely used QACs has been increasingly reported^24^. Similarly, triclosan has been criticized for its environmental persistence, endocrine-disrupting potential, and photodegradation into toxic dioxins^25,26^.

Photocatalytic pigments such as titanium dioxide (TiO₂) represent another antimicrobial strategy. Upon ultraviolet (UV) activation, TiO₂ generates reactive oxygen species (ROS) that exhibit strong cytotoxic effects^27^. These ROS are capable of inactivating multidrug-resistant bacteria, with Gram-negative strains generally being more susceptible due to their thinner cell walls^28,29^.

Powder coating additives currently rely mostly on silver or nano silver. Some silver-free systems using nano zinc oxide^30^, nano titanium dioxide^31,32^, or bismuth compounds^33^ have been proposed. However, metal-based nanoparticles may cause aggregation, phase separation, and toxicological issues, necessitating surface modification^34^.

In contrast, natural antimicrobial agents have gained increasing attention in recent years. Chitosan, for example, has demonstrated promising performance when incorporated into coating formulations^35^. ε-Poly-L-lysine (ε-PL), a biodegradable and cationic peptide composed of 25–35 L-lysine residues^36^, is water-soluble, non-toxic, and produced trough microbial fermentation. It exhibits broad-spectrum antibacterial activity^37^. Applications of ε-PL have been reported in the preservation of aquatic food products^38^ and in biocompatible surface coatings^39^. Moreover, Hu et al.. developed ε-PL-modified titanium surfaces that were effective against multidrug-resistant bacteria^40^.

Yuan et al. prepared antibacterial montmorillonite (MMT) materials intercalated with ε-PL or its hydrochloride form, both exhibiting strong active against *E. coli* and *B. subtilis*^41^. Similarly, Liao et al. developed ε-PL/chlorhexidine/MMT multilayer coatings that were effective against *S. aureus*^42^. Hybrid films containing ε-PL, MMT, and gentamicin sulfate (GS) demonstrated controlled and stimuli-responsive antibiotic release^43^. However, ε- polylysine has not yet been investigated as an active component in powder coatings systems.

In the present study, ε-poly-L-lysine (ε-PL) was incorporated into epoxy-based powder coatings both in its pristine form and intercalated within montmorillonite (MMT), with or without aminododecanoic acid (ADA). The objective was to evaluate the influence of these bioactive additives on the antimicrobial, aesthetic, and mechanical performance of the coatings, thereby providing a sustainable, silver-free alternative for antimicrobial protection.

The selected active components were expected to exhibit substantially lower toxicity toward human tissues compared to silver. Both ε-polylysine^44^ and montmorillonite^45^ are well established in biomedical applications. For example, Cu and Ag nanoparticles (NPs) immobilized in montmorillonite have been reported to be less toxic than the free nanoparticles while maintaining comparable antimicrobial activity^46^(Table 1).

**Table 1:** List of acronyms used in this study.

Acronym	Full term	Description
ADA	Aminododecanoic acid	Co-intercalating agent improving compatibility and dispersion
DMA	Dynamic mechanical analysis	Technique for viscoelastic and mechanical characterization
DSC	Differential scanning calorimetry	Method to study curing behavior and thermal transitions
EDS	Energy-dispersive X-ray spectroscopy	Technique for elemental composition analysis
FTIR	Fourier-transform infrared spectroscopy	Analytical method for identifying chemical structure
MMT	Montmorillonite	Layered silicate used as a carrier for bioactive molecules
PLY	ε-Polylysine	Cationic biopolymer with antimicrobial properties
SEM	Scanning electron microscopy	Imaging technique for surface morphology
SKP	Scanning Kelvin probe	Surface analysis technique to map potential distribution
TGA	Thermogravimetric analysis	Technique to measure mass change with temperature
WCA	Water contact angle	Measure of surface wettability and hydrophobicity
CFU	Colony-forming units	Unit for quantifying bacterial reduction
*E. coli*	*Escherichia coli*	Gram-negative test bacterium
*S. aureus*	*Staphylococcus aureus*	Gram-positive test bacterium
PBS	Phosphate-buffered saline	Medium used for bacterial suspension and testing

## Experimental part

### Preparation of antimicrobial agents (AA) and powder coatings

Antimicrobial agents were prepared by intercalating ε-polylysine (PLY), with or without aminododecanoic acid (ADA), into sodium montmorillonite (MMT). The resulting modified clays were then incorporated into epoxy-based powder coating formulations and applied onto steel substrates under controlled conditions.

Detailed information on synthesis, formulation, application, and curing procedures is provided in the Supporting Information (Sections S1–S3). The compositions of the reference and functionalized powder coatings are summarized in Table 2.

**Table 2:** Qualitative/quantitative composition of the powder coatings.

Component/ Symbol of coating	epoxy resin, wt %	G-92, wt%	Benzoin	Byk 368P	PLY wt%	PLY/MMT wt%	PLY/MMT/ADA wt%
EP (reference sample)	83.5	15.0	0.5	1.0	-	-	-
PLY	82.0	14.5	0.5	1.0	2.0	-	-
PLY/MMT	82.0	14.5	0.5	1.0	-	2.0	-
PLY/MMT/ADA	82.0	14.5	0.5	1.0	-	-	2.0

### Measurements

Characterization methods included SEM, XRD, FTIR, TGA, DSC, DMA, SKP, profilometry, gloss, adhesion, hardness, and microbiological tests (EN ISO 22196).

Detailed descriptions of all procedures are provided in the Supporting Information (Sections S4–S6).

## Results and discussion

### Characterization of antimicrobial agents

**Fig. 1:**
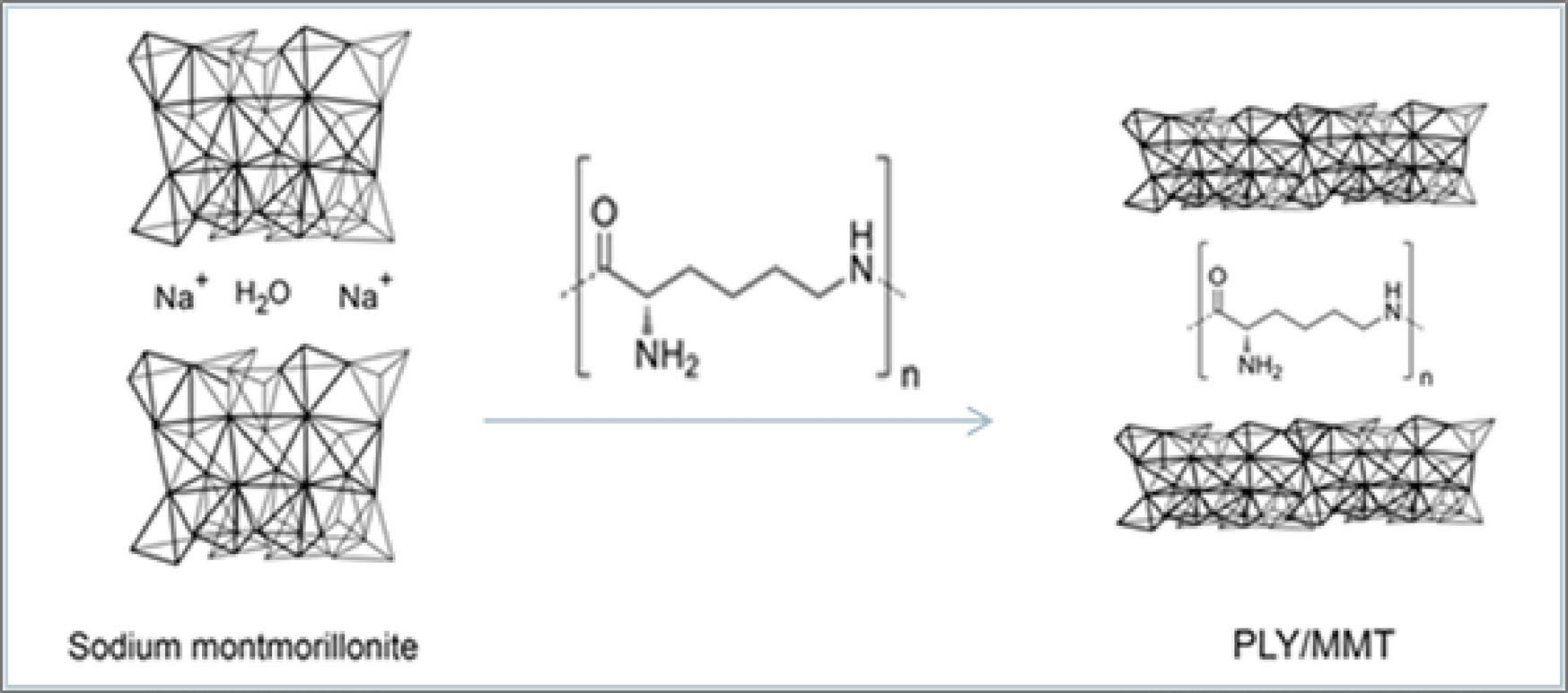
Imobilisation of ε-polylysine into sodium montmorillonite (Na⁺-MMT) to form biofunctional PLY/MMT nanohybrids with antimicrobial properties.

ε-Polylysine (PLY) was used to impart antibacterial functionality to the powder coatings, either in its protonated pristine form or after immobilization within montmorillonite (MMT) (Fig. 1). For the latter, two modification routes were examined: (i) intercalation of PLY from aqueous solution and (ii) co-intercalation with aminododecanoic acid (ADA).

Adding a second intercalant with a long alkyl chain, such as aminododecanoic acid (ADA), can facilitate PLY intercalation. The 12-carbon chain is believed to penetrate the interlayer space of montmorillonite (MMT), increasing the distance between adjacent layers. This effect is reflected in the XRD pattern of ADA-intercalated MMT, where the (001) reflection shifts to lower 2θ angles, indicating an interlayer expansion from 12.8 Å (pristine MMT) to approximately 20 Å. Immobilizing ε-polylysine in MMT is expected to reduce its leaching from the coating during prolonged moisture exposure.

**Fig. 2:**
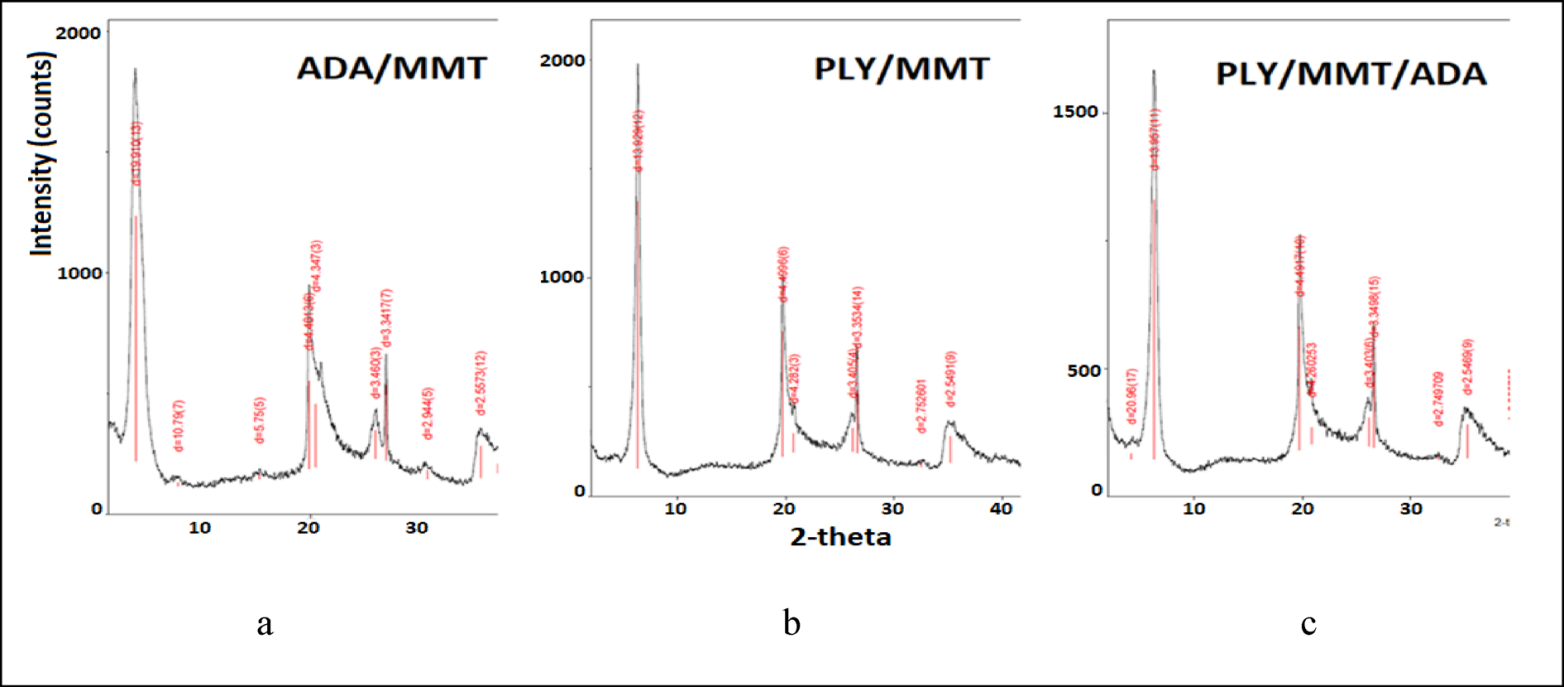
X-ray diffraction (XRD) patterns of (**a**) ADA/MMT, (**b**) PLY/MMT, and (**c**) PLY/MMT/ADA showing the (001) reflection shift upon intercalation.

The PLY- and/or ADA-modified MMTs were characterized using elemental analysis, X-ray diffraction (XRD), and BET surface area measurements. The corresponding results are summarized in Table 3 and illustrated in Fig. 2.

**Table 3:** Characteristics of components used in the study.

AA symbol	C [%]	H [%]	*N* [%]	C/*N* w/w	Interlayer distance d_001_ Å (XRD)	Specific surface area m^2^/g (BET)
PLY	39.87	8.024	15.00	2.66	-	-
MMT	-	-	-	-	12.8	29.67
ADA/MMT	18.30	3.40	2.20	8.32	19.91	12.73
PLY/MMT	7.11	2.033	3.00	2.37	13.9	20.19
PLY/MMT/ADA	7.88	2.167	2.81	2.80	14.0 (21)	21.91

Combustion elemental analysis revealed that both the carbon content and the C/N weight ratio were lower in PLY/MMT than in ADA/MMT, which can be attributed to the higher carbon content of the long-chain amino acid. The co-intercalated PLY/MMT/ADA showed only a slight increase in these values compared with PLY/MMT but differed substantially from ADA/MMT.

Considering similar BET surface areas and the (001) reflection positions in the XRD patterns, ε-poly-L-lysine likely represents the dominant intercalating species. A weak additional low-angle reflection at approximately 20 Å indicates that a fraction of long-chain amino acid molecules may also be partially incorporated between the MMT layers (Fig. 2).

To evaluate the thermal stability of the newly developed antimicrobial components under the conditions relevant to powder coating processing and curing, thermogravimetric analysis (TGA) was carried out. The corresponding results are provided in the Supporting Information (Section S7) and summarized in Table 4.

**Table 4:** TGA results of used components.

AA symbol	Decomposition temperature ranges /% wt. loss	DTG max	total % wt. loss at 700 °C
MMT	25–180/10 200–700/3	76.5 639	13
ADA	180–260 380–500	209 462	99
MMT/ADA	25–200/3 200–700/27	45 260, 297, 354	30
PLY	25–180/8 240–560/83	76 310,425,444	91
PLY/MMT	25–180/13 180–600/14	57 330, 470, 559	27
PLY/MMT/ADA	25–270 270–700	50/5 331, 436, 556	20

The first stage of thermal decomposition of the raw materials and intercalated montmorillonites involves desorption of water adsorbed within the aluminosilicate interlayers. The water content depends on hydrophobicity, being highest for PLY/MMT (13%) and lowest for MMT/ADA (3%). In sodium montmorillonite, the subsequent mass loss between 500 and 700 °C and is associated with clay dehydroxylation^47^.

Thermogravimetric analysis (TGA) of ε-poly-L-lysine revealed two main decomposition steps: 180–380 °C (65% mass loss) and above 380 °C (26%). Aminododecanoic acid (ADA) also decomposes in two steps — the first at approximately 180 °C (attributed to Hofmann elimination of β-H) and the second at around 400 °C, corresponding to the degradation of the organic residue^48^. PLY leaves a higher char residue (9%), whereas ADA exhibits an almost compelet mass loss (≈ 99%), which can be explained by its low molecular weight and high aliphatic carbon content.

PLY intercalation into MMT results in a shift of the DTG peaks toward higher temperatures, suggesting improved thermal stability. The mass loss above 380 °C (≈ 10%) was greater than that in the 180–380 °C range (≈ 4%). In contrast, ADA shows lower decomposition temperatures after intercalation, consistent with the findings of Liu et al., who attributed this behavior to the catalytic effect of montmorillonite’s surface acidity on amino acid degradation^49^.

The co-intercalation product PLY/MMT/ADA exhibited thermal behavior like that of PLY/MMT but yielded a slightly higher char residue (≈ 80%) compared with ADA/MMT (≈ 70%) or PLY/MMT (≈ 73%).

The powder coating process was carried out at 115 °C, followed by curing at 130 °C. TGA results indicated that only moisture release occurs within this range. Above 130 °C, the mass remained stable up to approximately 180 °C, confirming that the antimicrobial components were thermally stable under the conditions relevant to powder coating processing.

The chemical structure of the obtained antimicrobial agents was examined by Fourier transform infrared (FTIR) spectroscopy, with spectra provided in the Supporting Information (Section S8). The FTIR spectra of PLY/MMT and PLY/MMT/ADA were dominated by characteristic montmorillonite bands at approximately 1000 cm⁻¹ (Si–O stretching vibrations) and by a weaker band at around 3620 cm⁻¹ (O–H stretching vibrations) (Figs. S8.1b and S8.1c)^50^. Additional absorption peaks at around 3230 cm⁻¹, 2935 cm⁻¹, 1670 cm⁻¹, and 1550 cm⁻¹ originate from polylysine (Fig. S8.1a) and correspond to the vibrational modes of amide groups and protonated –NH₃⁺ side chains^51,52^. These spectral features support the presence of protonation of nitrogen atoms in polylysine, enabling electrostatic interactions between –NH₃⁺ groups and negatively charged sites on montmorillonite (MMT). The presence of protonated ammonium groups is considered a key factor influencing the antibacterial activity of polylysine.

**Fig. 3:**
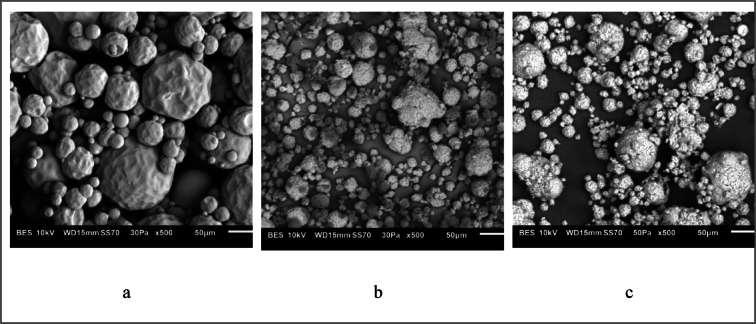
SEM morphology of antimicrobial additives: (**a**) polylysine (PLY), (**b**) polylysine immobilized on sodium montmorillonite (PLY/MMT) and (**c**) polylysine immobilized on aminedodecanoic acid intercalated on MMT (PLY/MMT/ADA).

The morphology of the antimicrobial additives was subsequently examined by scanning electron microscopy (SEM) (Fig. 3).

The morphology of the ε-poly-L-lysine–based antimicrobial additives varied depending on the treatment method. Pure polylysine particles showed a nearly spherical shape with a slightly developed surface, and their size ranged from approximately 5 to 100 μm. Polylysine particles immobilized on sodium montmorillonite displayed a more structured surface and a smaller particle size, ranging from about 1 to 40 μm. In contrast, PLY immobilized on aminododecanoic acid–modified MMT (PLY/MMT/ADA) showed a comparable particle size to the unmodified variant but exhibited a more pronounced surface roughness. Additionally, these particles tended to form agglomerates on the surface of larger powder particles.

### Powder coatings characterization

Powder coatings were prepared using ε-poly-L-lysine (PLY), either in its pristine form, intercalated into montmorillonite (PLY/MMT), or co-intercalated with aminododecanoic acid (PLY/MMT/ADA). The intercalation process aimed to enhance the dispersibility of the modifier within the powder matrix, improve compatibility with the polymer network, and increase washing resistance.

The coatings were cured at 130 °C for 10 min, during which epoxy groups from the resin reacted with the phenolic curing agent E011 to form a crosslinked network. The resulting coatings were evaluated for their visual appearance, mechanical performance, and antibacterial activity. Defect-free surfaces were obtained, showing no signs of orange peel, cratering, or pinholes. The corresponding properties are summarized in Table 5.

**Table 5:** Specifications of coatings properties.

Symbol of coating		EP	PLY	PLY/MMT	PLY/MMT/ADA
Roughness EN ISO 12,085	R_a_, µm R_z_, µm	0.82 ± 0.02 5.01 ± 0.16	0.21 ± 0.02 1.38 ± 0.12	0.36 ± 0.02 2.75 ± 0.17	0.18 ± 0.03 0.95 ± 0.18
Gloss for the angle of 60 deg EN ISO 2813	GU	72.3 ± 1.2	104.9 ± 1.5	74.2.1 ± 0.9	99.8 ± 2.6
Scratch resistance EN ISO 1518	g	400	400	300	300
Relative hardness EN ISO 1522	-	0.80 ± 0.02	0.90 ± 0.02	0.83 ± 0.03	0.86 ± 0.03
Adhesion to the steel EN ISO 2409	0-best 5-worst	0	0	0	0
Cupping ISO 1520	mm	8.70 ± 0.01	4.02 ± 0.01	8.74 ± 0.01	9.35 ± 0.01
Water contact angle EN 828	Deg	89.0 ± 1.2	87.7 ± 1.1	89.1 ± 1.2	88.2 ± 1.4
Reduction of *E. coli* EN ISO 22,196	Log reduction % reduction	Ref. sample: log total number of cells 4.32 ± 0.021	4.80 ± 0.022 99.998 ± 0.001	2.52 ± 0.061 99.698 ± 0.013	0.15 ± 0.082 29.77 ± 0.039
Reduction of *S. aureus* EN ISO 22,196	Log reduction % reduction	Ref. sample: log total number of cells 4.77 ± 0.011	2.57 ± 0.055 99.733 ± 0.091	0.33 ± 0.027 52.881 ± 0.170	0.15 ± 0.079 30.00 ± 0.245

The surface roughness of coatings was determined using the average parameters R_a_ and R_z_. The obtained values were characteristic of smooth coatings. However, the reference sample exhibited higher roughness (R_a_ = 0.82 ± 0.02 μm, R_z_ = 5.01 ± 0.16 μm) compared to the modified coatings.

The incorporation of ε-poly-L-lysine led to a reduction in surface roughness, indicating increased coating homogeneity. In contrast, modification with polylysine intercalated into montmorillonite (PLY/MMT) resulted in a slight increase in roughness relative to the PLY coating. This effect can be attributed to reduced homogeneity caused by differences in interfacial interactions between the resin and the more hydrophilic MMT particles. The addition of polylysine immobilized on aminododecanoic acid–modified MMT (PLY/MMT/ADA) again reduced the surface roughness, which is explained by the presence of the hydrophobic ADA chains enhancing compatibility with the epoxy matrix.

**Fig. 4:**
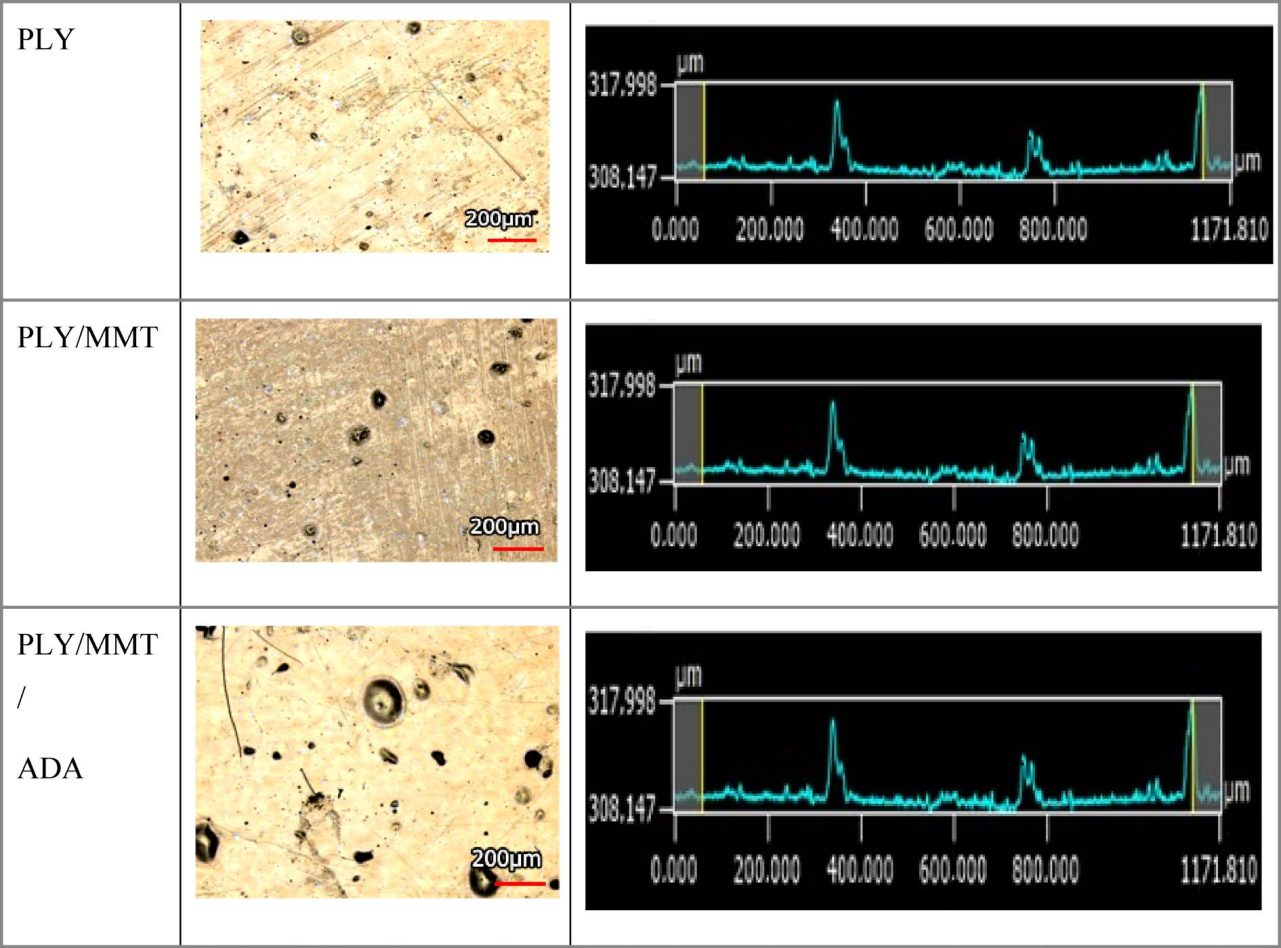
Surface images and profiles of selected coatings obtained by laser scanning microscopy (LSM). The visualizations show the surface morphology and roughness variations for the PLY, PLY/MMT, and PLY/MMT/ADA coatings.

In addition to stylus profilometry (Table 5), complementary roughness measurements were performed using laser scanning microscopy (LSM) (Fig. 4). This technique provides high-resolution, three-dimensional visualization of the surface topography and quantitative roughness parameters such as Ra and Rz. The results for selected coatings were as follows:

- PLY: Ra = 0.680 μm, Rz = 9.32 μm.
- PLY/MMT: Ra = 2.932 μm, Rz = 42.35 μm.
- PLY/MMT/ADA: Ra = 2.579 μm, Rz = 43.91 μm.

These results confirm the trends observed by stylus profilometry: coatings modified with nanofillers such as MMT and ADA showed increased surface roughness compared with the unmodified PLY coating, likely due to the presence of dispersed filler particles that increase the topographical variation.

Laser scanning microscopy (LSM) surface analysis showed that the unmodified coating (PLY) had the lowest roughness (Ra = 0.68 μm, Rz = 9.32 μm). In contrast, the nanocomposite coatings PLY/MMT (Ra = 2.93 μm, Rz = 42.35 μm) and PLY/MMT/ADA (Ra = 2.58 μm, Rz = 43.91 μm) exhibited higher roughness values. These results suggest that the incorporation of MMT and ADA leads to a more textured surface morphology, likely due to particle agglomeration and the formation of micro-protrusions. Such features may influence barrier properties and adhesion behavior of the coatings. The 3D LSM images further illustrate differences in surface features and filler distribution between the composite coatings.

In summary, LSM-based roughness analysis complements stylus profilometry by providing spatially resolved, microstructural information. The combination of both techniques provides additional insight into the relationship between surface topography and coating performance.

The gloss of the coatings correlated inversely with their surface roughness — smoother coatings exhibited higher gloss values. All coatings demonstrated good scratch resistance. Samples containing ε-poly-L-lysine (PLY) showed scratch resistance comparable to that of the reference coating. In contrast, coatings containing montmorillonite (MMT) exhibited slightly lower scratch resistance, which may be related to the inherently low hardness of MMT (1.5 on the Mohs scale). The incorporation of polylysine was associated with the epoxy-based reference coating, likely due to the presence of stiffer molecular chains and additional intermolecular hydrogen bonds. Furthermore, during the crosslinking process, epoxy groups from the resin may react with amino groups of polylysine, forming additional crosslinks that could contribute to the increased hardness^53^. For MMT-containing coatings, hardness also increased relative to the reference but remained slightly lower than that of the PLY coating, consistent with the contribution of the rigid lamellar MMT structure. Coatings containing polylysine showed reduced cupping resistance, which may result from the influence of its linear molecular structure on the tribological behavior of the crosslinked epoxy matrix. In contrast, MMT-containing coatings exhibited higher cupping resistance due owing to the presence of strong Si–O (1096 kJ mol⁻¹) and Al–O (957 kJ mol⁻¹) bonds in MMT, compared with the weaker C–C (347 kJ mol⁻¹), C–H (415 kJ mol⁻¹), and C–O (360 kJ mol⁻¹) bonds in the epoxy matrix^54^. All coatings showed good adhesion to the steel substrate, likely due to the presence of polar functional groups such as secondary hydroxyls formed during the curing reaction between the epoxy resin and the phenolic curing agent. These hydroxyl groups may promote electrostatic interactions with the steel surface, supporting improved adhesion. The PLY-containing coating exhibited a lower contact angle, consistent with its more hydrophilic character.

The antibacterial performance data show that the PLY-containing coating exhibited excellent activity against *E. coli* (99.998% reduction) and slightly lower activity against *S. aureus* (99.733%). These results suggest that PLY is more effective against Gram-negative bacteria, possibly due to structural differences in their cell walls. When immobilized in MMT, PLY retained high activity against *E. coli* but showed markedly reduced efficacy against *S. aureus*. Co-intercalation with aminododecanoic acid further decreased antibacterial activity, resulting in approximately a 30% reduction for both bacterial strains.

To better understand the influence of aminododecanoic acid (ADA) on antimicrobial effectiveness, a coating containing 2 wt% ADA (as its phosphoric acid salt used in the co-intercalation process) was prepared. This coating showed no measurable antibacterial activity against *E. coli* and achieved approximately 69% reduction of *S. aureus* colonies.

In the study by Togashi et al.^55^, long-chain fatty alcohols with fewer than seven or more than seventeen aliphatic carbons exhibited little or no bactericidal activity against *S. aureus*, whereas dodecanol showed relatively strong antibacterial effects. Similarly, Zhang et al.^56^ reported that medium- and long-chain fatty acids demonstrate antimicrobial activity against *S. aureus*. In our unpublished studies, the co-intercalation of ADA with montmorillonite and an oligomeric peptide led to increased antibacterial activity of the modified MMT against Gram-positive bacteria, while the effect was less pronounced against Gram-negative species. However, in the present study, no clear beneficial effect of ADA was observed on the bactericidal activity toward either *S. aureus* or *E. coli*. For organic biocides intercalated in montmorillonite, antimicrobial performance is influenced by several factors, including the concentration and mode of biocide immobilization (ion exchange, van der Waals forces^57^ as well as electrostatic and hydrophobic interactions^58^. Incorporation of the MMT intercalation compound into the polymer coating, followed by curing, may further complicate the assessment of how these structural factors affect biocidal performance toward specific bacterial strains. Interestingly, montmorillonite containing silver dispersed in an epoxy/polyester powder coating showed no antimicrobial effect against *E. coli*, which was attributed to insufficient wetting of the polymer coating, hindered silver ion diffusion^59^.

**Fig. 5:**
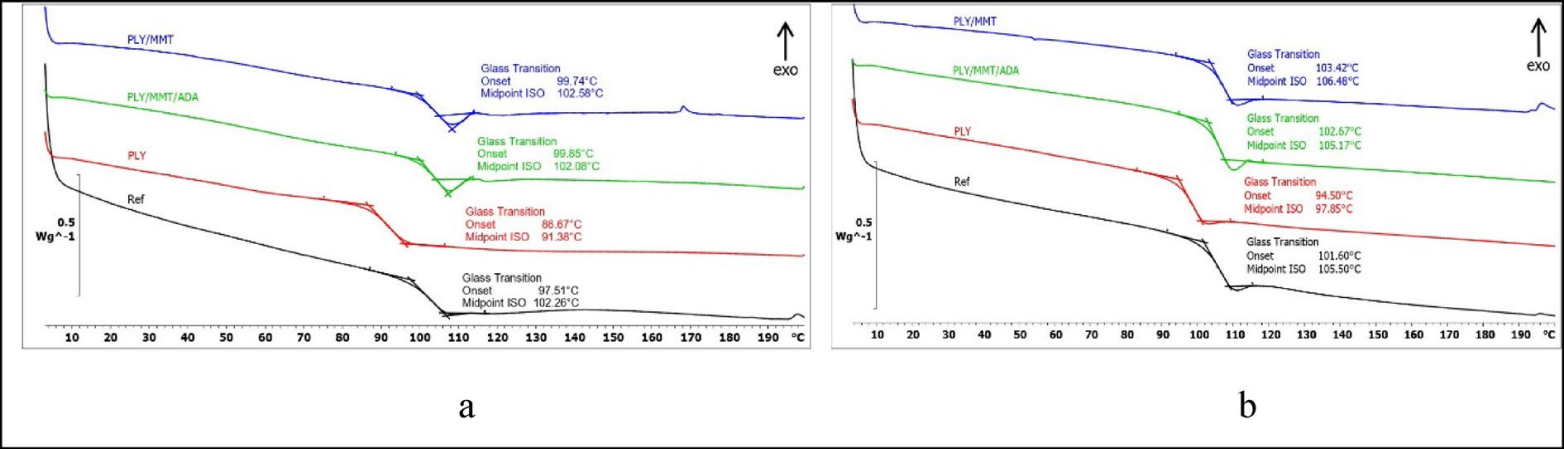
DSC curves recorded during (**a**) the first heating, (**b**) the second heating.

**Fig. 6:**
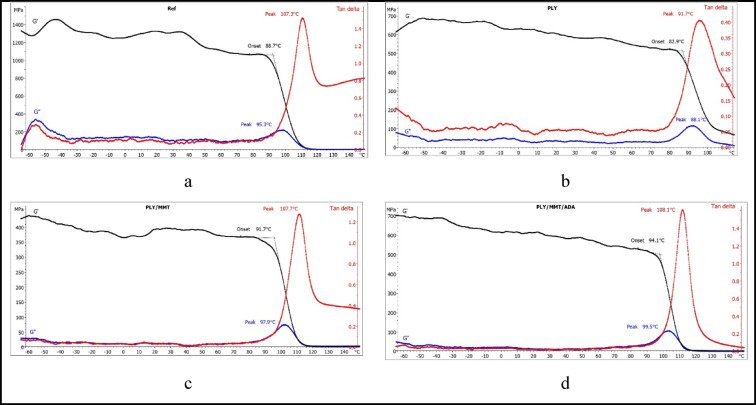
DMA curves of the samples: (**a**) reference sample without microbial additives, (**b**) sample with polylysine (PLY), (**c**) with polylysine immobilized in montmorillonite (PLY/MMT), (**d**) with polylysine immobilized in montmorillonite with aminododecanoic acid (PLY/MMT/ADA).

The effect of the antimicrobial additives on the thermal behavior of the powder coatings during controlled heating was investigated using differential scanning calorimetry (DSC) and dynamic mechanical analysis (DMA) (Figs. 5 and 6). The DSC curves recorded during the first heating cycle showed a glass transition accompanied by relaxation phenomena. The glass transition temperature (*T*g) of the epoxy reference coating (EP) was determined to be 102.3 °C (Fig. 5a). For coatings containing 2 wt% of the biocidal compositions PLY/MMT and PLY/MMT/ADA, the *T*g values were 102.6 °C and 102.1 °C, respectively, indicating that the addition of these hybrid fillers did not significantly affect the transition temperature of the epoxy binder.

In contrast, the addition of 2 wt% ε-poly-L-lysine (PLY) alone led to a decrease in *T*g by approximately 10.9 °C compared with the reference sample (Fig. 5a). This shift may indicate specific interactions between PLY and the epoxy resin. Chen et al.^60^ reported that PLY can interact with C–O groups in epoxy matrices through NH functionalities, an effect that may contribute to the observed glass transition behavior. Similarly, Shukla et al.^61^ found that the glass transition temperature of microbial crystalline poly(ε-L-lysine) is 88 °C. It is worth noting that lysine and lysine-based (amino-functionalized polyester) systems exhibit tunable thermal properties — such as glass transition and melting temperature — which increase with the molecular weight (Mn) of the amino-functionalized polyester.

The glass transition temperature of the reference coating, determined from the DSC curve recorded during the second heating cycle, was 105.5 °C, compared with 106.5 °C for the PLY/MMT coating and 105.2 °C for the PLY/MMT/ADA coating (Fig. 5b). The slight variations in the measured *T*g values can be attributed to the removal of thermal history and internal stresses during the first heating stage. The coating containing 2 wt% PLY exhibited a lower *T*g of 97.9 °C. The smaller difference in *T*g between the PLY-containing coating and the reference in the second heating cycle suggests a more ordered binder structure after thermal conditioning.

The dynamic mechanical analysis (DMA) revealed that the glass transition was the predominant transition observed during testing. The peak of the mechanical loss factor (tan δ) was used to determine the glass transition temperature (*T*g). For the reference coating, *T*g was determined to be 107.3 °C (Fig. 6a). Very similar values were obtained for the PLY/MMT and PLY/MMT/ADA coatings—107.7 °C and 108.1 °C, respectively (Fig. 6c and d). The lowest *T*g value of 91.7 °C was recorded for the coating containing 2 wt% PLY (Fig. 6b).

This sample also showed the lowest tan δ peak intensity, suggesting enhanced damping (attenuation) capability. The other samples showed comparable tan δ intensities. Analysis of the storage modulus (*E*) revealed that the coatings underwent relaxation and intramolecular reorganization of the epoxy (EP) matrix at the initial stage of the testing, followed by vitrification. At higher temperatures, the samples entered a more elastic state, as evidenced by the reduced *E* values The influence of MMT and ADA in the PLY/MMT/ADA formulation was particularly noticeable: this coating exhibited the highest storage modulus among all samples (approximately 700 MPa, compared to 550 MPa for the reference, 600 MPa for PLY, and 450 MPa for PLY/MMT). These results suggest greater energy storage capacity and higher stiffness of the PLY/MMT/ADA coating under dynamic shear deformation.

**Fig. 7:**
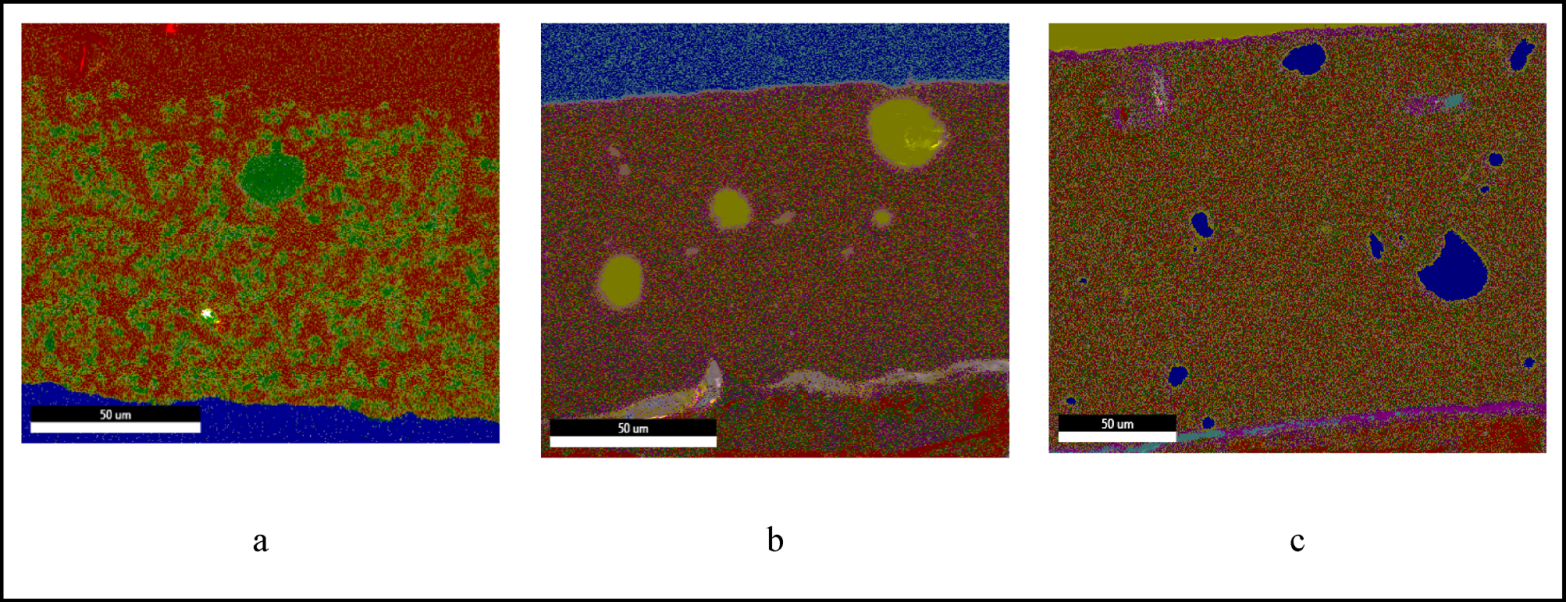
EDS microscopy images and elemental mapping of epoxy powder coatings: (**a**) PLY, (**b**) PLY/MMT, and (**c**) PLY/MMT/ADA. Quantitative elemental compositions corresponding to these mappings are summarized in Table 6.

To assess the elemental distribution and homogeneity of the antimicrobial coatings, SEM/EDS mapping and quantitative analysis were performed for three formulations: PLY, PLY/MMT, and PLY/MMT/ADA (Fig. 7; Table 6).

The elemental composition and distribution of the antimicrobial powder coatings were evaluated by EDS spectroscopy and are summarized in Table 6.

**Table 6:** Elemental composition (in weight%) of powder coatings containing polylysine (PLY), polylysine immobilized in montmorillonite (PLY/MMT), and polylysine immobilized in montmorillonite modified with aminododecanoic acid (PLY/MMT/ADA), as determined by EDS spectroscopy.

Element	Weight%_ PLY	Weight%_ PLY/MMT	Weight%_PLY/MMT/ADA
CK	21.81	75.54	23.52
OK	18.36	3.95	28.74
FeI	1.34	0.33	1.88
AlK	2.05	0.03	9.43
SiK	0.33	0.29	23.49
AuM	20.98	19.84	10.45
NK	0.27		0.08
KK			0.26
NaK			0.15
BaI	29.08		
SK	5.77		
MgK		0.01	1.47

For the PLY coating, the EDS spectrum showed a high carbon content (~ 74.6 wt%) together with notable amounts of oxygen (11.8 wt%), nitrogen (2.2 wt%), and sulfur (5.4 wt%). The presence of nitrogen and sulfur is consistent with the chemical composition of ε-poly-L-lysine and the epoxy resin. Elemental mapping indicated a generally homogeneous distribution of components, suggesting uniform film formation. Minor traces of sodium, aluminum, and silicon were detected, likely originating from the substrate or incidental contamination.

In the PLY/MMT formulation, the successful incorporation of montmorillonite was evidenced by a marked increase in silicon (13.4 wt%) and aluminum (2.6 wt%), accompanied by a slight decrease in carbon content (~ 75.6 wt%) due to the inorganic nature of the filler. The oxygen content was also higher, reflecting contributions from the silicate framework. Elemental maps revealed localized montmorillonite agglomerates with higher density areas, although overall dispersion remained relatively uniform. The consistent nitrogen signal suggests that ε-poly-L-lysine remains well distributed throughout the matrix.

For the PLY/MMT/ADA coating, the oxygen (21.6 wt%) and nitrogen (4.2 wt%) contents were higher, consistent with the additional functional groups introduced by aminododecanoic acid (ADA). The carbon content decreased to ~ 31.5 wt%, while the silicon and aluminum levels remained comparable to those of the PLY/MMT coating, suggesting a stable montmorillonite fraction. Elemental mapping revealed localized clusters that may correspond to ADA-rich domains or phase-separated regions. These inhomogeneities were also visible as contrast variations in the SEM micrographs. Such segregation may influence both the electrochemical surface behavior (as observed by SKP measurements) and the accessibility of antimicrobial agents^62,63^.

**Fig. 8:**
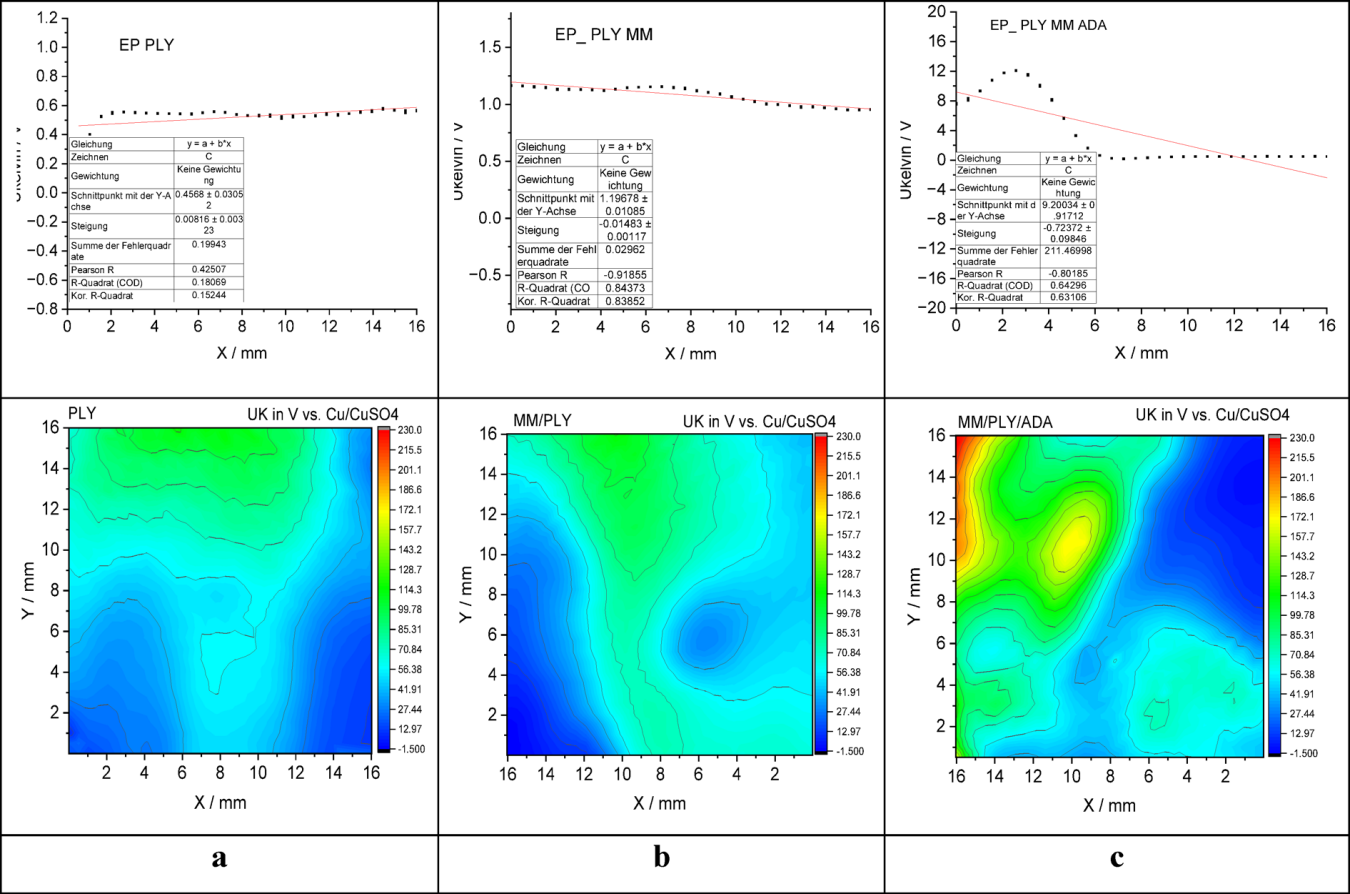
Surface potential distribution of antimicrobial powder coatings measured by Scanning Kelvin Probe (SKP): (**a**) PLY, (**b**) PLY/MMT, and (**c**) PLY/MMT/ADA.

The surface potential distribution of the antimicrobial powder coatings was subsequently analyzed using scanning Kelvin probe (SKP) measurements to evaluate the influence of ε-poly-L-lysine and its combinations with montmorillonite (MMT) and aminododecanoic acid (ADA) on electron-transfer characteristics at the coating surface (Fig. 8).

**Fig. 9:**
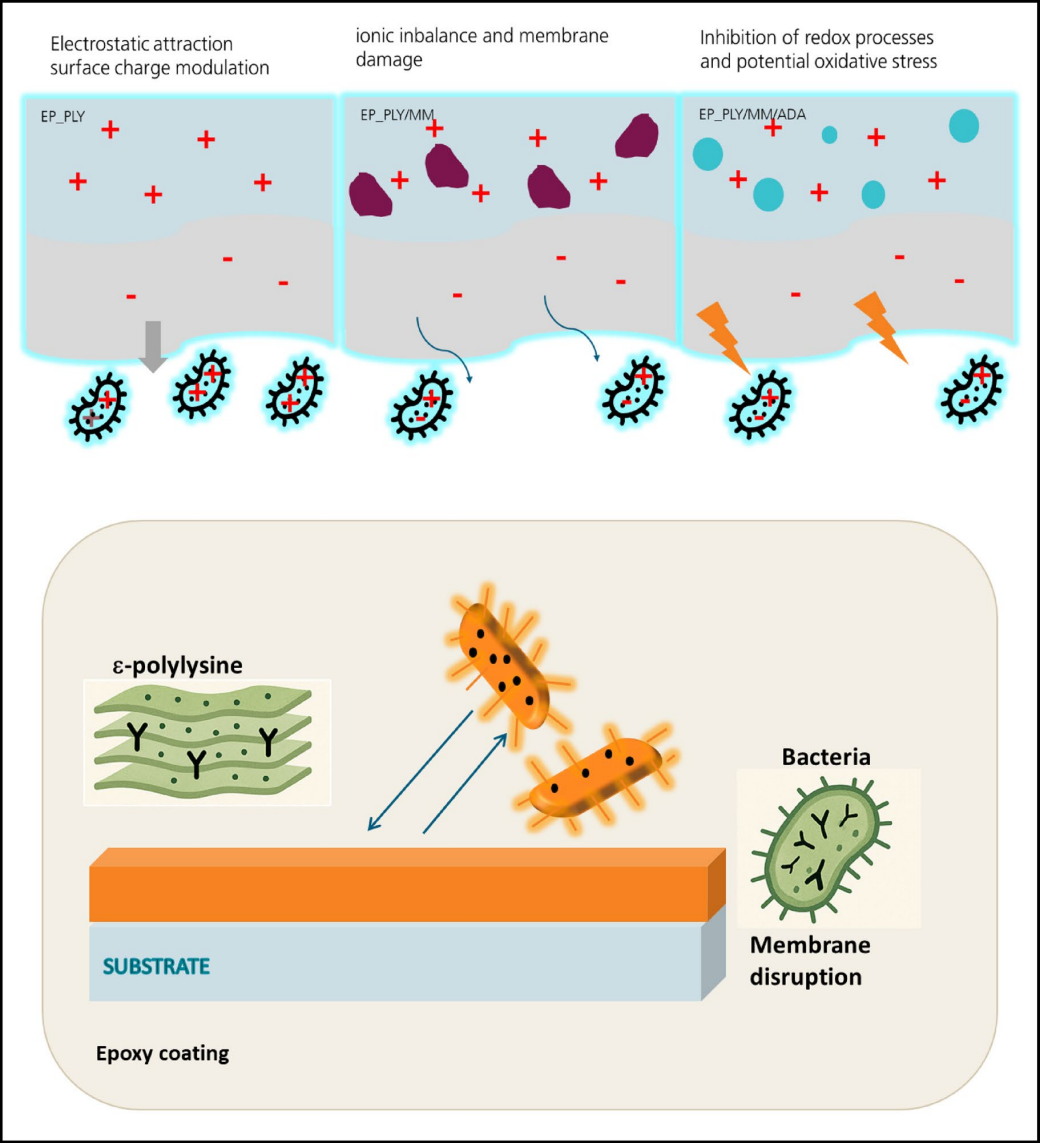
Schematic illustration of proposed antibacterial mechanisms of PLY/MMT systems, created by the authors using Microsoft PowerPoint.

For the PLY coating, the surface potential ranged from approximately 0.46 V to 0.56 V, displaying a slight positive gradient across the scanned area (Fig. 8a). This suggests a mildly electron-enriched surface, which may facilitate initial oxygen reduction, followed by the formation of a positively charged electrical double layer that suppresses further redox activity.

In contrast, the PLY/MMT coating exhibited a distinct negative gradient in surface potential, decreasing from about 1.3 V to 1.0 V across the surface (Fig. 8b). This behavior suggests that interactions between ε-poly-L-lysine and the montmorillonite matrix induce electron depletion and enhanced band bending, thereby reducing surface reactivity, particularly toward oxygen adsorption and reduction.

The most pronounced effect was observed for the PLY/MMT/ADA coating (Fig. 8c). The surface potential showed a sharp gradient ranging from above + 12 V to below + 2 V, with localized charge accumulation evident in the lower-left region of the map. This pronounced potential inhomogeneity may result from phase separation or the formation of microdomains. The strong lateral potential differences and steep voltage drop indicate significant spatial variation in electron distribution, which could reduce electron mobility. Such effects may contribute to the diminished antimicrobial performance observed for this formulation.

Overall, these findings suggest that the surface electrochemical landscape of antimicrobial powder coatings can be modulated through additive composition. While pure ε-poly-L-lysine appears to enhance surface electron availability, its combination with layered fillers and co-additives introduces structural and electronic complexity that can impair surface reactivity.

A comparison of SKP results with EDS data reveals a correlation between chemical homogeneity and surface potential stability:

- PLY: homogeneous structure, stable surface potential.
- PLY/MMT: moderate agglomeration, downward potential trend.
- PLY/MMT/ADA: visible inhomogeneities, sharp potential gradients and localized charge accumulation.

These observations suggest that, although additive combinations such as MMT and ADA can improve certain physical properties, they may also promote microphase separation, which may influence charge transport and potentially reduce antimicrobial efficiency.

### Washing resistance of antibacterial additives

To evaluate the resistance of the powder coatings to leaching of the antibacterial components, changes in water conductivity upon contact with the cured coatings were monitored over time. Conductometric analysis of the washing resistance of coatings containing PLY, PLY/MMT, and PLY/MMT/ADA is provided in the Supporting Information (Sect. 9). For the coatings containing ε-poly-L-lysine alone, a marked increase in conductivity was observed after 5–7 days of immersion, indicating gradual dissolution of the polymer containing ionic groups. Immobilization of PLY within the montmorillonite structure significantly reduced leaching for up to three weeks, after which a moderate rise in water conductivity was detected.

In contrast, the co-intercalated PLY/MMT/ADA system exhibited no measurable increase in conductivity compared to the biocide-free reference coating, even after 25 days of immersion, confirming the stabilizing effect of amino acid modification on the antimicrobial additive.

### Mechanism of antimicrobial action

The antimicrobial activity of powder coatings containing ε-poly-L-lysine and its combinations with montmorillonite (MMT) and aminododecanoic acid (ADA) can be explained by a set of physicochemical interactions occurring at the coating–microorganism interface.

According to Nigmatullin et al.^64^, intercalation compounds of montmorillonite with cationic polymers exhibit two principal modes of antimicrobial activity: (i) migration of the biocidal species toward the surface, and (ii) contact-killing of bacteria through interactions with immobilized cationic groups. Because the diffusion of the biocide is relatively slow, the contact mechanism is generally considered to be dominant.

Based on previous reports and the newly obtained SKP and EDS data, three major mechanisms are proposed to explain the antimicrobial behavior of the studied coatings (Fig. 9):

1. Electrostatic attraction and surface charge modulation:

In contrast, incorporation of montmorillonite (PLY/MMT) altered the surface potential. The negatively charged layered structure of the clay induced electron depletion and a decrease in surface potential. This redistribution of surface charge may reduce electrostatic attraction between the coating and bacterial cells, thereby weakening the interaction and diminishing antimicrobial efficiency.

(2) Ionic imbalance and membrane damage:

In the PLY/MMT/ADA system, SKP contour plots revealed pronounced potential gradients and regions of elevated surface charge. Such gradients may create localized electric fields that disturb osmotic balance across bacterial membranes, leading to ion and water leakage, membrane rupture, and subsequent cell death. This interpretation is supported by EDS mapping, which indicated partial phase separation and heterogeneity of ADA-rich domains, consistent with local charge accumulation and field inhomogeneity.

(3) Inhibition of redox processes and potential oxidative

Electron-transfer reactions, particularly oxygen reduction at the coating surface, are influenced by the surface potential. For the PLY coating, a moderately positive potential may allow limited redox activity. However, the pronounced potential drop observed in the PLY/MMT/ADA system suggests an electron-depleted surface state, which could suppress oxygen reduction. Such electron deficiency may hinder redox reactions and interfere with microbial respiration processes. Conversely, localized high-potential zones may facilitate the generation of reactive oxygen species (ROS), leading to oxidative damage and metabolic disruption in bacterial cells.

In summary, the antimicrobial effectiveness of ε-poly-L-lysine–based coatings appear to depend strongly on additive chemistry, surface potential, and spatial charge distribution. A homogeneous, moderately positive potential—as observed for the PLY coating— seems most favorable for electrostatic interaction and antimicrobial performance. In contrast, composites containing MMT and ADA introduce surface inhomogeneity, which may reduce biocidal efficiency by impairing charge transfer and local electron balance.

## Conclusions

In this study, silver-free antimicrobial epoxy powder coatings incorporating the cationic biopolymer ε-poly-L-lysine (PLY) were developed and comprehensively characterized. PLY was employed in three forms: as a pure additive, intercalated within montmorillonite (PLY/MMT), and co-intercalated with aminododecanoic acid (PLY/MMT/ADA) to modulate compatibility and leaching behavior.

Physicochemical analyses (FTIR, XRD, TGA, DSC, DMA, SEM/EDS and SKP indicate that the structure and dispersion influence thermal and mechanical properties, as well as surface electrochemical behavior. Among the investigated systems, the PLY-only coating showed a homogeneous surface morphology, a stable positive surface potential, and high antibacterial reduction under laboratory conditions (ISO 22196) against *E. coli* and *S. aureus*, together with a water contact angle of approximately 85°.

Within the limitations of this laboratory-scale, the use of ε-poly-L-lysine—a biodegradable and renewable biopolymer—suggest a promising and sustainable strategy for developing silver-free antimicrobial powder coatings. Future work should focus on evaluating cytotoxicity and long-term environmental durability to further validate the safety and real-world applicability of these materials. Although such aspects were beyond the scope of this study, they represent essential directions for continued research.

## Limitations and outlook

This work was conducted on laboratory-scale samples and short-term antibacterial tests. Consequently, long-term durability under relevant environmental/industrial exposures, leaching over extended periods, and cytotoxicity/biocompatibility were not assessed here. Future studies should (i) verify real-world performance (e.g., abrasion, cleaning cycles, humidity/temperature cycling), (ii) quantify long-term antimicrobial efficacy and any potential release of active species, (iii) evaluate cytotoxicity and regulatory aspects for use near medical or food-related environments, and (iv) address scale-up and process robustness on industrial coating lines. These steps are essential to confirm safety and applicability beyond the present laboratory conditions.

## Supplementary Information

Below is the link to the electronic supplementary material.

Supplementary Material 1

## Data Availability

All data generated or analysed during this study are included in this published article and its supplementary information files. Additional data is available from the corresponding author on reasonable request.
