# Supplementary material for: Antimicrobial activity of silver free powder coatings based on biocomponents
Source: Sci Rep. 2025 Dec 2;15:43021. doi: 10.1038/s41598-025-30166-3 (PMC12675507; doi:10.1038/s41598-025-30166-3)
Supplement: Supplementary file 1 — Supplementary Material 1 [file 41598_2025_30166_MOESM1_ESM.docx]

**Supporting Information**

*Antimicrobial activity of silver free powder coatings based on biocomponents*

Katarzyna Krawczyk^a*^, Barbara Pilch-Pitera^b^, Michał Kędzierski^c^, Małgorzata Zubielewicz^d^, Ewa Langer^d^, Sebastian Jurczyk^d^, Grażyna Kamińska-Bach^d^, Katarzyna Daszykowska^b^, Leszek Komorowski^e^, Katarzyna Bieniek^c^, Marta Przybysz-Romatowska^c^, Michael Hilt^a^

^a^ [Fraunhofer Institute for Manufacturing Engineering and Automation IPA](https://www.ipa.fraunhofer.de/en.html), Pigments and Coatings Allmandring 37, 70569 Stuttgart, Germany

^b^ Faculty of Chemistry, Department of Polymers and Biopolymers, Rzeszow University of Technology, ul. Powstańców Warszawy 6, 35-959 Rzeszów, Poland

^c^ Łukasiewicz Research Network – Industrial Chemistry Institute, ul. Ludwika Rydygiera 8, 01-793 Warsaw, Poland

^d^ Łukasiewicz Research Network – Institute of Polymer Materials,M. Skłodowskiej-Curie 55, 87-100 Toruń, Poland

^e^ Road and Bridge Research Institute, ul. Instytutowa 1, 03-302 Warszawa, Poland

Corresponding author: [katarzyna.krawczyk@ipa.fraunhofer.de](mailto:katarzyna.krawczyk@ipa.fraunhofer.de)

**S.1. Preparation of antimicrobial agents (AA)**

Raw materials:

- ε -polylysine (PLY), >95% (Marknature)
- Sodium montmorillonite nanoclay (MMT) (Sigma-Aldrich)
- Hydrochloric acid (36%) (Chempur).

Polylysine (16 g) was dissolved in water acidified with HCl (in a stoichiometric amount relative to the amino groups of PLY) at 50°C until a homogeneous mixture was obtained. Subsequently, MMT-Na (50 g) was added, and the resulting suspension was heated for 2 hours at 70°C. It was set aside until the next day, after which the solution was decanted from the precipitate, which was washed twice with approximately 1.5 L of water at 50°C, followed by 1-1.5 L of water at room temperature. After the precipitation settled, the excess water was decanted; the MMT/PLY product was filtered under reduced pressure and dried in a vacuum dryer at 50°C until a constant mass was achieved. Similarly, polylysine (8 g) and aminododecanoic acid (ADA, 11.7 g) were co-intercalated into MMT (50 g) using an equimolar ratio of both modifiers based on amino groups, resulting in the product PLY/MMT/ADA.

**S.2. Preparation of powder coatings**

Raw materials:

- Epoxy resin: E011, E.E.W. 650-850 (Sarzyna Chemical, Poland)
- Catalysed phenolic curing agent: G-92, softening point 80 - 100 °C (Worlee, Germany)
- Degassing agent: Benzoin (Aldrich, Switzerland)
- Flow control agent: Byk 368P (Byk, Germany).
- Antimicrobial agents: ε-polylysine, PLY immobilized on sodium montmorillonite (PLY/MMT), or polylysine immobilized on aminedodecanoic acid intercalated montmoryllonite (PLY/MMT/ADA)

The qualitative and quantitative composition of the powder coatings were given in Table 1. All raw materials were weighed and mixed. Then the mixture was grinded and extruded in a co-rotating twin screw mini extruder (EHP 2 x 12 Sline from Zamak, Poland). The extrusion temperature was as follows: zone I-60 °C, zone II-90 °C, zone III-100 °C, and adapter-115 °C. The screw’s rotational speed was 150 rpm. After extrusion, the cooled extrudates were pulverized, and sieved in a 100 μm sieve.

Table. 1. Qualitative/quantitative composition of the powder coatings

| **Component/**  **Symbol of coating** | **epoxy resin, wt %** | **G-92, wt%** | **benzoin** | **Byk 368P** | **PLY**  **wt%** | **PLY/MMT**  **wt%** | **PLY/MMT/ADA**  **wt%** |
| --- | --- | --- | --- | --- | --- | --- | --- |
| EP (reference sample) | 83.5 | 15.0 | 0.5 | 1.0 | - | - | - |
| PLY | 82.0 | 14.5 | 0.5 | 1.0 | 2.0 | - | - |
| PLY/MMT | 82.0 | 14.5 | 0.5 | 1.0 | - | 2.0 | - |
| PLY/MMT/ADA | 82.0 | 14.5 | 0.5 | 1.0 | - | - | 2.0 |

**S.3. Application on the substrate and cross-linking of the coatings**

The powder coatings were applied to the matt steel substrate (Q-Panels R-36) and glass plates using a CORONA spray gun (PEM X-1) controlled by an EPG Sprint X unit (Wagner, Alstatten, Switzerland). The coated samples were then cured at 130 °C for 15 minutes.

**S.4. Characterisation of antimicrobial agent**

Elemental analysis of carbon, hydrogen and nitrogen was performed using a UNICUBE/Rapid OXY Cube elemental analyzer (ELEMENTAR Analysensysteme, Langenselbold, Germany).

Thermogravimetric analysis (TGA) was conducted using a thermobalance TGA/SDTA 851^e^ (Mettler-Toledo). The measurement parameters were as follows: temperature range from 25 to 600°C, heating rate of 10 °C/min., nitrogen atmosphere, gas flow 50 cm^3^/min, sample weight ~5 mg, 150 μl open alumina pans.

The morphology and microstructure of the antimicrobial additives were characterized using a scanning electron microscope (SEM, JEOL JSM-6010LV) in backscattered electron shadow imaging mode in low vacuum.

Powder X-ray diffraction (XRD) spectra were recorded on a MiniFlex600 powder diffractometer, Rigaku, with the following parameters: radiation: CuKα1, ƛ=1.54056 Å, scanning range 2θ: 2 – 80°.

The analysis of the specific surface area (BET) was carried out using the isothermal nitrogen sorption and desorption method using the Micromeritics TriStar II 3020 analyzer.

**S.5. Characterisation of powder coatings**

A Mar Surf PSI profilometer was used to measure the roughness of cured powder coatings in accordance with series of EN ISO 12085.

A micro-TRI-gloss µ tester from BYK-Gardner GmbH, was used to determine the gloss of the cured powder coatings in accordance with EN ISO 2813:2014. The gloss was measured at 60° angle.

The scratch resistance test was carried out using a manual Clemen tester from Elcometer, in accordance with the EN ISO 1518-1:2019.

Coatings adhesion to the steel surface was evaluated by a cross-cut test according to EN ISO 2409:2022.

König Pendulum tester from BYK-Gardner GmbH was used to measure the relative hardness of the cured powder coatings according to EN ISO 1522:2008.

The measurement of the water contact angle was carried out by the side drop method using an optical goniometer by Data Physic, model OCA 15, in accordance with the EN 828:2013.

Antibacterial activity on the coating surfaces was evaluated according to EN ISO 22196:2011 against *E. coli* and *S. aureus* bacteria. A bacterial suspension (1 mL, 1–5 × 10⁸ cells/mL) was mixed with 100 of agar suspension at 45 ± 2 ℃. The test~~ed~~ coatings were placed in Petri dishes and moistened with saline solution using sterile, cotton plugs. Then, 0.5 ml of agar suspension with inoculated bacterial culture was applied on the costing surface. This allowed the formation of a layer of "pseudo-biofilm" with a height not exceeding 1 mm. After the suspension solidified, the coatings were incubated for 24 hours at 37 ℃ in a high-humidity incubator so that the agar suspension with the inoculated culture did not dry out. After incubation, both control and tested samples were transferred from the Petri dishes into sterile beakers, using tweezers sterilized in a burner flame. 4.5 ml of neutralizing broth was measured to each beaker ("A"), thus obtaining a dilution of 1 :10 based on the original inoculum. Beakers were sealed with parafilm and subjected to sonication and vortexing for 1 minute. Then a series of 10-fold dilutions were made: 4.5 ml of neutralizing broth were measured to 15 ml falcons, 0.5 ml of the solution containing bacteria from the samples was taken from the beakers ("A") and transferred to the first row of falcons ("B"), after thorough mixing, 0.5 ml of the solution was transferred from falcon "B" to "C", and then from "C" to "D". A final dilution of 1:10000 relative to the original inoculum was obtained. Inoculation on agar medium: Each of the Petri dishes containing the agar medium was divided into 3 parts, into which 25 μl of the appropriate solution were transferred and distributed evenly by means of sterile spreaders. The samples were incubated at 37℃ for 24 hours. After 24 hours of incubation, the number of bacterial colonies that had grown on the plates was counted and recorded. Reduction of bacteria was calculated as the difference between total number of cells grown on the reference sample (coating do not containing of antimicrobial agent) and number of cells grown on the tested sample (coating containing antimicrobial agent). The statistical study was based on three independed sample. Standard deviation (SD) of % cell number of each bacteria was calculated based on 3 samples. For calculation of % reduction deviation and Log reduction deviation a complete differential method was used.

A Scanning Kelvin Probe Microscopy (SKP) device from Anfatec was used to measure the potential distribution on the surface of cured powder coatings.

An Energy-dispersive X-ray spectroscopy (EDX) analysis was performed using a Helios NanoLab 600i dual-beam scanning electron microscope.

Quantitative surface feature analysis was carried out using a 3D Laser Scanning Microscope (Keyence).

Fourier-transform infrared spectroscopy (FTIR) was performed using a spectrometer Nicolet iS10 Thermo Scientific. ATR/diamond crystal technique was used to obtain spectra of the coating surface.

Differential scanning calorimetry (DSC) of the samples was performed using a model DSC 1 Mettler-Toledo (Greifensee, Switzerland). Samples of 7÷9 mg, in the form of discs of 5 mm diameter, taken form the free coatings, were heated from 0ºC to 200ºC at a rate of 20ºC min^-1^ and cooled at the same rate in a nitrogen atmosphere at a flow rate of 60 ml min^-1^. Samples were sealed in a 40 µL aluminum DSC crucibles. Glass transition temperatures were determined during the second heating runs. Data analysis of DSC results was performed using the supplied STAR^e^ Mettler-Toledo v.15 software.

To measure the storage modulus, loss modulus and tan *δ* of coatings the Dynamic-mechanical analysis (DMA) was used. Measurements were made using a DMA 861^e^ Mettler-Toledo (Greifensee, Switzerland). The samples, in the form of disc-shaped specimens taken from free coatings, were heated from -65ºC to 150ºC at a heating rate of 3ºC min^-1^. The shear mode was used to test the coatings with a displacement amplitude of 1 μm and a frequency of 1 Hz. Data analysis of the DMA results was performed using the supplied STAR^e^ Mettler-Toledo v.15 software.

**S. 6. Washing resistance of antibacterial additives**

The leachability of antibacterial additives from powder coatings was investigated by measuring the electrical conductivity of the aqueous solution at various time intervals. Coating samples of similar dimensions and mass were prepared and placed in laboratory containers. A specific volume of deionized water (60 ml) was added to each sample. The containers were tightly sealed and kept at room temperature.

Electrical conductivity was measured using a Microcomputer Conductivity Meter (Elmetron CC-551). Conductivity was recorded at designated time intervals—after 24 hours, 96 hours, 7 days, and so on. The samples remained immersed in water for a period of two months. The conductivity electrode was rinsed with deionized water before and after each measurement.

The study was conducted at room temperature (approximately 22–25°C), and temperature fluctuations were monitored, as the electrical conductivity of water is temperature-dependent.

**S. 7. TGA curves of antimicrobial additives**

| 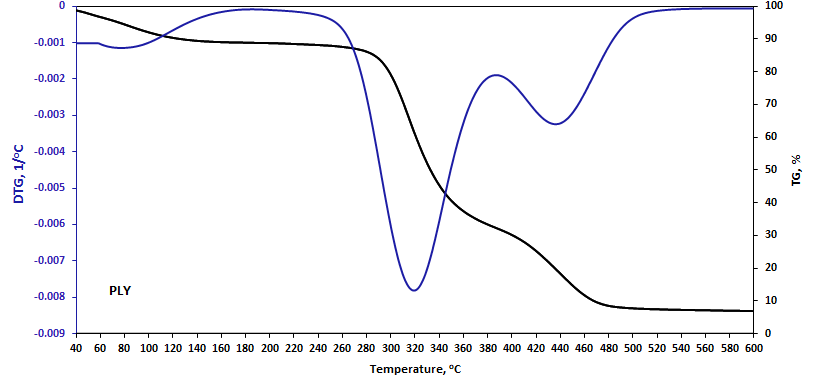 |
| --- |
| a |
| 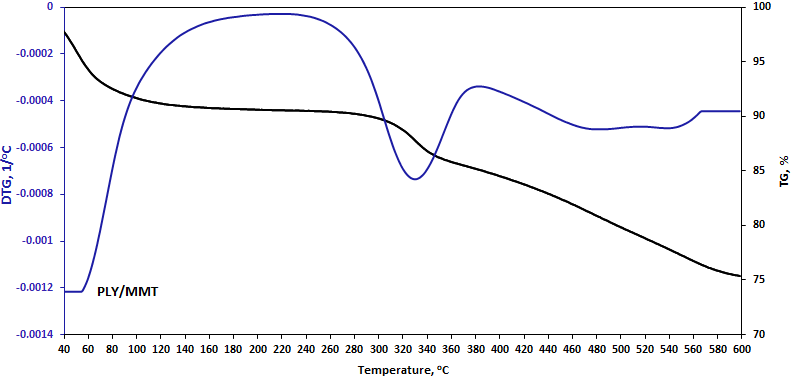 |
| b |
| 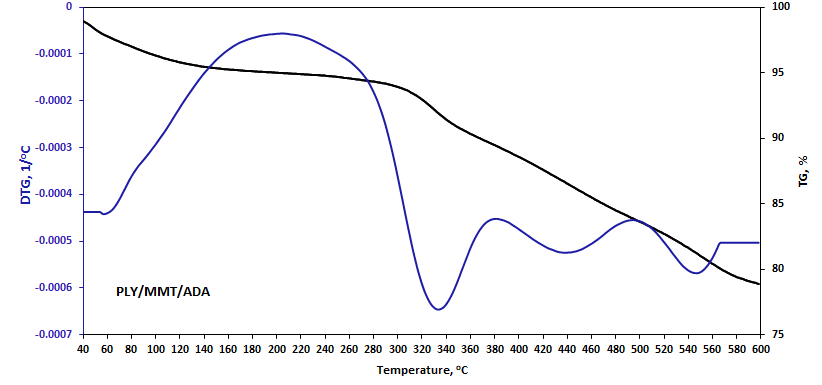 |
| c |

Fig. S.7.1. TGA curves of antimicrobial additives: a) polylysine (PLY), b) polylysine immobilized on sodium montmorillonite (PLY/MMT) and c) polylysine immobilized on aminedodecanoic acid intercalated on MMT (PLY/MMT/ADA)

**S. 8. FTIR spectra of antimicrobial additives**

a


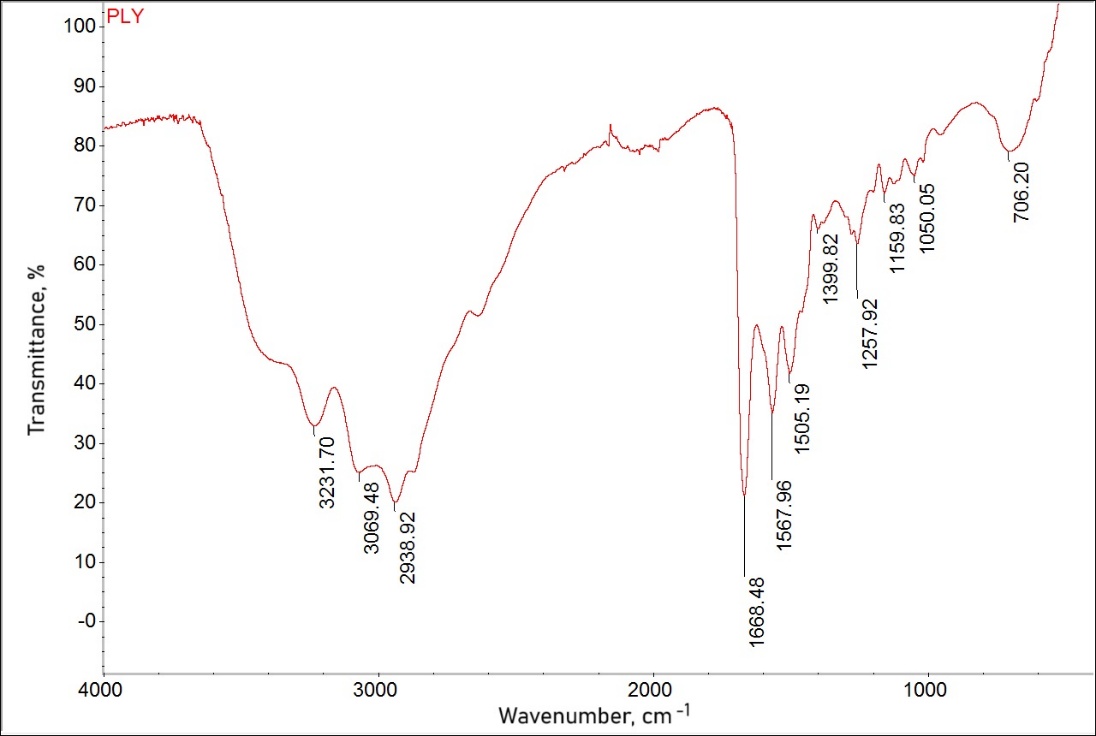


b


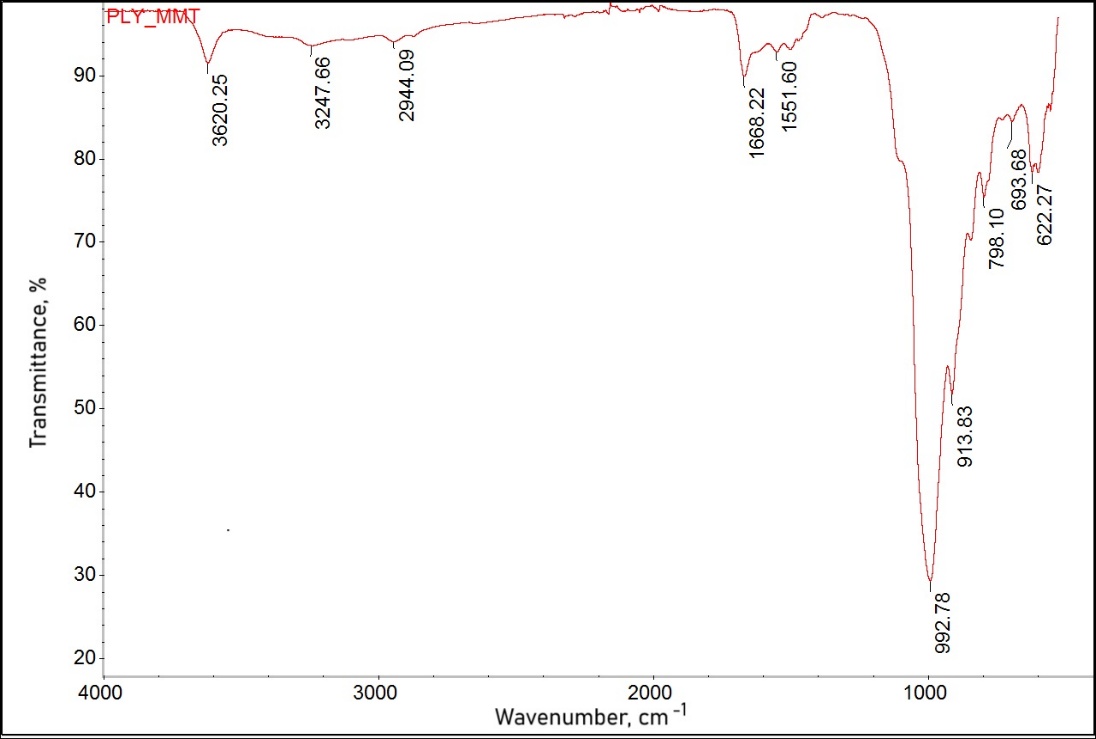


c


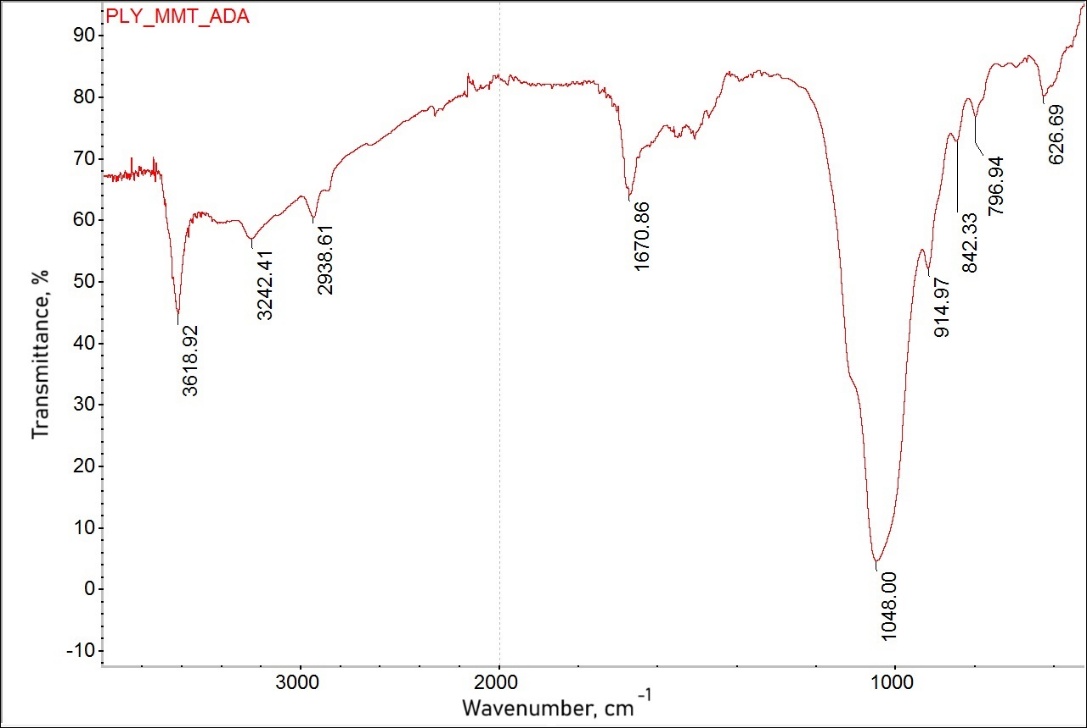


Fig. S.8.1. FTIR spectra of microbial additives: a) pure polylysine (PLY), b) its immobilization product in montmorillonite (PLY/MMT), c) its immobilization product with aminododecanoic acid in montmorillonite (PLY/MMT/ADA)

**S. 9. Conductometric analysis of the washing resistance of powder coatings containing PLY, PLY/MMT, and PLY/MMT/ADA**


Fig. S.9.1. Conductometric analysis of the washing resistance of powder coatings containing PLY, PLY/MMT, and PLY/MMT/ADA. The graph shows the changes in water conductivity over time, reflecting the leaching behavior of antimicrobial agents.
